# New Recovery Strategies in Motor and Cognitive Functions, before, during and after Home-Confinement COVID-19, for Healthy Adults and Patients with Neurodegenerative Diseases: Review

**DOI:** 10.3390/jcm11030597

**Published:** 2022-01-25

**Authors:** Manuela Bacanoiu, Mircea Danoiu, Mihnea Marin, Mihai Robert Rusu, Ligia Rusu

**Affiliations:** 1 Sport Medicine and Physical Therapy Department, University of Craiova, 200585 Craiova, Romania; manuela.bacanoiu@edu.ucv.ro (M.B.); mircea.danoiu@edu.ucv.ro (M.D.); mihai.rusu@edu.ucv.ro (M.R.R.); 2 Industrial Engineering Department, University of Craiova, 200585 Craiova, Romania; mihnea.marin@edu.ucv.ro

**Keywords:** pandemic COVID-19, neurodegenerative diseases, physical activity, public health strategies, healthy lifestyle behaviors, wellbeing, telerehabilitation

## Abstract

Distancing and confinement at home during the Coronavirus Disease 2019 (COVID-19) outbreak has led to worsening of motor and cognitive functions, both for healthy adults and for patients with neurodegenerative diseases. The decrease in physical activity, the cessation of the intervention of the recovery and the social distance imposed by the lockdown, has had a negative impact on the physical and mental health, quality of life, daily activities, as well as on the behavioral attitudes of the diet. The purpose of this paper was to evaluate the impact of decreasing physical activity and the affected emotional status in healthy adults and patients with neurodegenerative diseases in conditions imposed by the stay at home mandate of COVID-19, along with new interventions, such as telemedicine and telerehabilitation. These interventions include online surveys carried out in multi-languages, semi-structured interviews, intervention smartphones and interventions through online platforms, for instance: Google, WhatsApp, Twitter, ResearchGate, Facebook and LinkedIn. For this study, we selected original papers that were intensively processed using characteristics co-related with physical activity, mental wellbeing, sleep quality, good eating behavior and healthy lifestyle. By searching the last two years of literature, our review presents and demonstrates the benefit of online technological interventions in lockdown, which promote physical exercise patterns and rehabilitation techniques, for healthy adults and patients with neurodegenerative diseases, and the need to develop new strategic directions and governmental measures, designed procedures and health services, which are expected to improve the quality of life, the progress of physical and cognitive functions, mental health and wellbeing for all.

## 1. Introduction

A novel coronavirus named severe acute respiratory syndrome coronavirus 2 (SARS-CoV-2), and the disease called COVID-19, detected in China in December 2019, managed to affect over 200 countries in just 6 months, reporting over 10 million illnesses and over half a million deaths. The World Health Organization (WHO) declared a global pandemic in March 2020, thus causing worldwide concern. Therefore, all societies affected by the SARS-CoV-2 infection have gradually declared social distancing and home-confinement to prevent the spread of the new infection. According to Tondo et al. [1], the COVID-19 pandemic generates substantial changes in routine activities, restrictions of movement and has a considerable impact on people’s psychological and cognitive levels. Furthermore, it creates challenges for the healthcare system. The main problems in dementia during this pandemic period are related to an increase of sufferers of cognitive impairment [1].

Under these conditions, the most drastic quarantine measures in human history were imposed [2]. This situation, imposed to slow the spread of COVID-19, has had negative effects on the quality of life, psychosocial and emotional behavior of individuals, even whether apparently healthy or with various associated diseases.

Due to social isolation and lack of direct communication, changes in emotional status have been reported, with the appearance of emotional disorders by way of feelings of loneliness, decreased personality (e.g., frustration, boredom, delusions, inadequate supplies) and distrust of peers [3,4,5].

Infection with SARS-CoV-2, which has caused severe acute respiratory syndrome and other distress for multiple organ failure, has affected a substantial number of patients with other comorbidities (such as Myasthenia Gravis, Vascular dementia) associated with neurodegenerative diseases, such as Parkinson’s disease (PD), Alzheimer’s disease, Frontotemporal dementia and Lewy body disease. Lack of therapeutic interventions, limiting access to specialized treatments, decreasing physical activity and help from caregivers and family during this period has aggravated functional abilities, cognitive impairments and behavioral disturbances [6].

During the isolation at home imposed by the COVID-19 pandemic, the needs for social assistance intervention increased, putting pressure on professional healthcare and home caregivers for patients with dementia [5]. Therefore, the imposed home confinement considerably restricted access to social and health services, which led to a worsening of behavioral and neuropsychological symptoms for them [6]. At the same time, a decrease in physical activity and limit of activity daily living (ADL), generate a change of lifestyle and life quality.

From this point of view, the promotion of a mental health and wellbeing lifestyle, involves continuing physical training. This means supporting movement skills, improving gait, increasing muscle strength and endurance, and prevents the progression of the disease in mild cognitive impairments (MCI). The challenge identified in the literature is that the COVID-19 outbreak has limited access to these activities due to isolation at home [7]. Hence, other interventions, such as aerobic exercises and home dance training had to be implemented to counteract the progressive deficiencies of patients with PD [8,9].

In addition to worsening motor and cognitive functions, the COVID-19 quarantine also impaired sleep quality, eating behavior affected mental wellbeing and induced depressive symptoms [10].

In the context of the COVID-19 outbreak, the need for modern digital therapies and those that are delivered remotely as well as their intervention at home have proven to be effective in the rehabilitation of patients with PD and other neurodegenerative disease. This could decrease the effect of motor skills, gait pattern, neuropsychiatric impairments and classic symptoms, such as tremor, bradykinesia, postural instability and freezing of gait [11]. The aim of the paper is to carry out a literature analyses regarding how the lockdown and physical activity influence motor and cognitive function, based on evaluation of the impact of decreasing physical activity, and the affected emotional status of healthy adults and patients with neurodegenerative diseases and associated comorbidities, such as Myastenia and Vascular dementia, in conditions imposed by COVID-19. The aim of the literature analysis includes a review of how the new interventions, such as telemedicine and telerehabilitation, could improve or maintain a healthy status, based on multi-language online surveys, semi-structured interviews and interventions on smartphones delivered through online platforms, for instance, Google, WhatsApp, Twitter, Research Gate, Facebook and LinkedIn. In our paper, we highlight the state of the art about what is written to date on the COVID-19 pandemic impact through the lives of healthy people and neurodegenerative disease patients.

Three questions guided the literature review: which physical activity has been chosen; what is the impact of the lockdown and decrease of physical activity under motor and cognitive aspects; and what is the impact of the interventions through telerehabilitation and telemedicine?

## 2. Materials and Methods

### 2.1. Selection Criteria

We searched in PUB MED electronic database the most relevant studies, using as keywords: “COVID-19 lockdown”, “wellbeing lifestyle”, “physical activity”, “neurodegenerative diseases”, “healthy policies” and “telerehabilitation”. A literature search was conducted on 1 September 2021. The sorting of the papers was conducted in accordance with the PRISMA flow diagram [12]. In our paper, we selected the most relevant articles, which used modern digital methods and online surveys imposed by the global pandemic COVID-19 lockdown. The articles included the patients with motor and cognitive impairments from neurodegenerative diseases, who were deprived for a long period of time by planned physical exercise or of accessing specialized social assistance services. Additionally, we included the articles focused on healthy adolescents/adults involved in planned physical exercise during the lockdown. The considered publications covered the last 2 years, since the entire world was affected by the imposed lockdown in this time period. We considered only materials published in the English language.

### 2.2. Selection Strategy

In this synthesis, several aspects were taken into account: (1) individuals in the study groups with neurodegenerative diseases who had varying degrees of motor and non-motor impairments (some studies also included comorbidities such as Myasthenia and Vascular dementia); (2) randomized clinical trials with a large number of participants of both sexes were evaluated; (3) physical interventions and semi-structured interviews were conducted through online digital platforms; (4) PA (physical activity) consisted of planned exercises of low, moderate or vigorous intensity, which were applied both to healthy people and to patients with confirmed neurodegenerative impairments (the alteration of the emotional status) during the period of isolation at home; (5) considerable emphasis was placed on ADL, these being the most publicized during the COVID-19 lockdown; (6) both experimental and control groups were included in the study; (7) motor and cognitive evaluation of participants was performed using different tools for assessing the levels of disorder; (8) finally, the interventions through telerehabilitation and telemedicine were evaluated and guidelines were issued in the elaboration of new remote technological intervention strategies as well as the involvement of decision makers in health policies.

Exclusion criteria for these studies were: comorbidities other like recent surgery or physical participation in other public health services.

### 2.3. Affiliation of Collecting Significant Data

The electronic PUB MED database from the last 2 years was accessed using the keywords referring to the periods of home confinement imposed by SARS-CoV-2 outbreak. Articles were searched that referred to neurodegenerative diseases with motor and cognitive disorders; strategies for wellbeing of physical and mental life as well as remote rehabilitation through stimulating physical activities to increase the quality of life.

In this sense, 65 of the most representative papers from the PUB MED electronic database were selected. The papers focused on received interventions at home during the restrictions imposed by the COVID-19 pandemic, through online digital platforms or semi-structured interviews, regarding physical exercise programs, development daily household activities, online interviews regarding physical and mental state of the participants or eating habits approached during this period. After the elimination of duplicate papers, 64 studies remained to be scanned, and of these, another 16 papers that did not refer to neurodegenerative diseases were eliminated. Of the 48 full-text clinical trials and articles assessed for eligibility, 30 were excluded for the following reasons: 12 studies were systematic reviews, 1 paper was a case study, 6 papers were abstract studies, 7 studies were not free to access and 4 papers did not refer to physical activity. Thus, in the end, only 18 studies that addressed healthy adults and patients with: Parkinson’s disease, Alzheimer’s disease, Lewy body disease, Frontotemporal dementia and associated diseases (Vascular dementia, Myasthenia gravis), will be discussed for qualitative synthesis.

### 2.4. Model Quality Assessment

Several variables were used in the selection and extraction of data: (1) author and date of publication, classification of disease stages and changes in their evolution through motor and cognitive instruments, (2) pattern of physical therapies (intensity, frequency and types of physical activity), performed by individuals at home under the conditions imposed by SARS-CoV-2 infection, (3) intervention of digitally accessed platforms and questions from specialists and staff trained in specialized remote health services, and answers of participants regarding physical and emotional status, (4) methods used, results, discussions and conclusions from each paper.

## 3. Results

**Figure 1:**
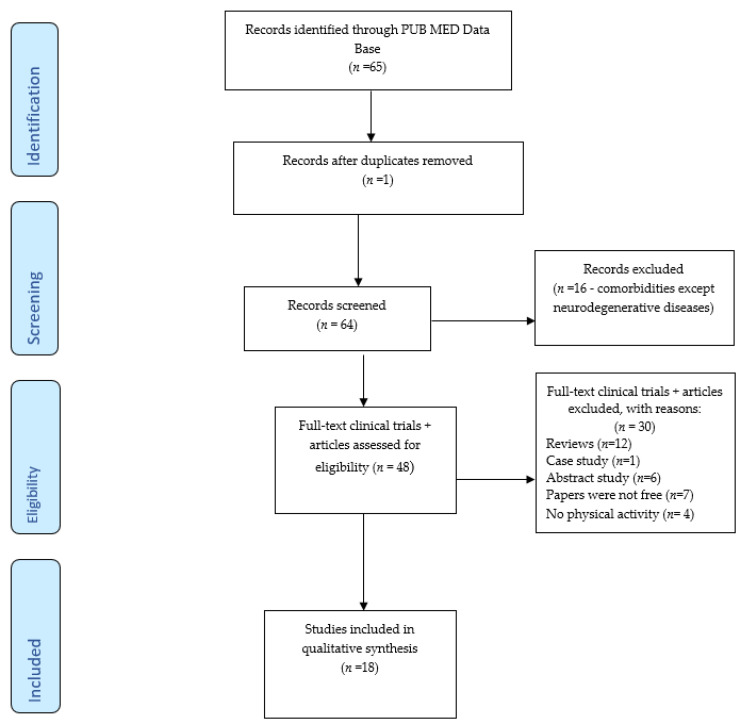
Prisma 2009. Flow diagram.

The graphical representation of the research dynamics is illustrated in Figure 1 of the PRISMA 2009 flow diagram [12]:

### 3.1. Tools/Variables Which Quantify Motor and Emotional Status in Healthy Adults during Home Confinement

Participation in social life or active integration in community activities improves everyone’s mental health, increases self-control and self-efficacy by demonstrating psychological and social wellbeing [13].

The major concern in lockdown was to better quantify the decrease in physical activity and its negative impact of cognitive function and life quality.

Through semi-structured electronic studies and interviews launched on online platforms by specialized organizations, the participants’ answers regarding mental wellbeing, the appearance and development of depressive symptoms as well as the comparison of situations before and during home confinement were requested.

The most relevant instruments for these purposes were: the Short Warwick–Edinburgh Mental. Wellbeing Scale (SWEMWBS), which assessed mental wellbeing [14] with a score between 7–35, and the superior shows considerable mental wellbeing; the Short Mood and Feelings Questionnaire (SMFQ), which demonstrated a depression measure where a high score means worsening psychic symptoms; the Short Life Satisfaction Questionnaire for Lockdowns (SLSQL), which quantifies the wellbeing of life [15]; the Short Social Participation Questionnaire for Lockdowns (SSPQOL) [15]; the social participation of individuals was quantified by the International Physical Activity Questionnaire Short Form (IPAQ-SF), which suggests participation in physical activity [15]; the Pittsburgh Sleep Quality Index (PSQI) [15], a tool that monitors sleep quality; the Short Diet Behaviors Questionnaire for Lockdowns (SDBQL), the scale that monitors eating habits [2]; the Short Technology use Behaviors Questionnaire for Lockdowns (STBQL), which provides demographic information and which allows psychological and social support [16]; and the Short Life Satisfaction Questionnaire for Lockdowns (SLSQOL) [16], framed as a score between 3 and 21, is a scale that measures the degree of satisfaction on the life of the respondents during the period of isolation at home, and the lowest scores appreciate a high degree of dissatisfaction in terms of active participation in social life. Decreasing socialization and communication with friends, neighbors, lack of visits to the respondents’ homes or to other relatives of their families, changed mental wellbeing and altered emotional status with increased anxiety, loneliness or sadness [12] are all considered here.

In the four studies [13,14,15,16] with 1047 participants each, using ECBL (Effects of Home Confinement on psychosocial health status and multiple lifestyle behaviors- COVID-19), an electronic survey was delivered on Google platforms, such as mail, WhatsApp, Facebook, ResearchGate, Twitter, LinkedIn, translated in the several languages, questions were administered about lifestyle, daily activities, diet, rest and mental state of participants before and during home confinement. Individuals were about 54% female and 46% male and the percentages by geographical areas were as follows: 40% Africa, 36% Asia, 21% Europe and 3% others.

For instance, in one of the four papers, the most significant tools used were: *SWEMWBS* who assessed the functioning of mental wellbeing with a score range between 7–35, so that the low values represented a low mental wellbeing and the higher ones appreciated a high functioning of the psychic status and SMFQ scale, which measures degree of depression (0–26). Thus, a total score greater than (˃12) indicated altered emotional status that indicate the presence of depression.

In another study that used the platform of ECLB COVID-19, monitoring was carried out on physical training through evaluation activities daily living (ADL), exercise class or gym class home-based. The basic tools used were SSPQOL who evaluated the social participation before and during home confinement, so that the 14 items in the questionnaires were based on the active participation of the respondents in social life. The range of scale was established between 15–70, so the higher scores, the more active the participation in social life in various ways was. Another variable, SLSQOL, used a questionnaire with answers and evaluated satisfaction life, and the score between 3–21 estimated the degrees of satisfaction or dissatisfaction. The lower scores showed different levels of disagreement and upper scores translated to being extremely satisfied. The other tools used were *SWEMWBS*, SMFQ, IPAQ-SF and PSQI, that estimated decreasing mental wellbeing, impairing sleep quality, changes in physical activity in the sense of decreasing it, or altering emotional status, with serious psychological consequences [14].

Another paper that used the questionnaire delivered through ECLB COVID-19, discussed IPAQ-SF guidelines, the variable, which quantified physical exercises of different intensity, estimating the time spent weekly a physical training, including time spent standing. Several categories of physical activities were performed, such as vigorous physical exercises with aerobic exercises or fast bicycling, moderate physical exercises meaning carrying light loads or cycling at normal speed, walking training and finally the time spent in the last 7 days sitting. In this sense, answers were requested for two sets of questions related to the different categories of physical activities performed during seven days, one addressed to young people and adults able to perform vigorous and moderate PA and the second set was delivered to older adults who did walking training and spent time sitting. The second tool used was SDBQ-L, which assessed diet behavior before and during the home confinement period. The requests in the questionnaires addressed five issues: (1) unhealthy food, (2) snacking between meals or late at night, (3) excessive alcohol consumption, (4) overeating and (5) a high number of meals/day. The trend observed during isolation at home is to address unhealthy eating habits that lead to weight gain, impaired health and wellbeing [15].

The scores of variables SWEMWBS, SMFQ and SLSQL, were significantly altered during isolation at home imposed by pandemic restrictions by altering mental wellbeing, increasing depressive states, accentuate nervousness, disorders of sleep, or changes in emotional status with accentuate sadness, feelings of loneliness, uselessness and personal dissatisfaction [16]. The scores from the other tools, including IPAQ-SF, SDBQL, PSQI, STBQ-L, SSPQ-L and SLSQL, demonstrated psychosocial impact imposed by home confinement, and the importance for technology intervention involved in active and healthy lifestyle.

Another article studied the impact of home confinement on the wellbeing of mental health correlated with physical activities performed at home of different intensities, eating habits acquired during the isolation period but also with the quality of sleep in Arabian communities. The impairment of mental well-being was more pronounced in Arab women (53.9%) compared to men (46.1%). In terms of the quality of the diet obtained through questionnaires, the highest score means maintaining mental wellbeing through the use of good meals and food. The quality of sleep was assessed through a range between 0 and 21 points with PSQI scale, so that the maximum values were associated with sleep disorders during the period of isolation at home affecting mental status. For physical activity, which was evaluated through IPAQ with seven items, the questioning proved that in the case of performing moderate and vigorous physical activity (measured by the distribution metabolic equivalents minutes per week (MET), wellbeing mental is improves. Furthermore, it was associated with increasing aerobic physical exercise and endurance physical activity [17].

Increased depression, anxiety, sleep disturbances, disturbances of attention or psychological changes were discussed in another study conducted in adolescents, through the excessive use of social networks and dangerous exposures to them, such as cyber bulling, sexting in desire of communication and compression of social distancing during the quarantine of COVID-19.

However, there was a greater resistance of adolescents to external stressors as there was an effective alternative to promote socialization, minimize social distance, by using online technology that they mastered very well. Monitoring was performed by counting text and chat messages that were sent or received from online platforms during the lockdown. There were even very well maintained physical activities at home, promoted sustained periods of time or daily activities that improved the physical and culinary performance of the participants. Relationships between participants and other family members were greatly improved during this period, and eating habits were much healthier than during direct socialization [18].

#### Physical Activity in Healthy Adults during Home Confinement

**Figure 2:**
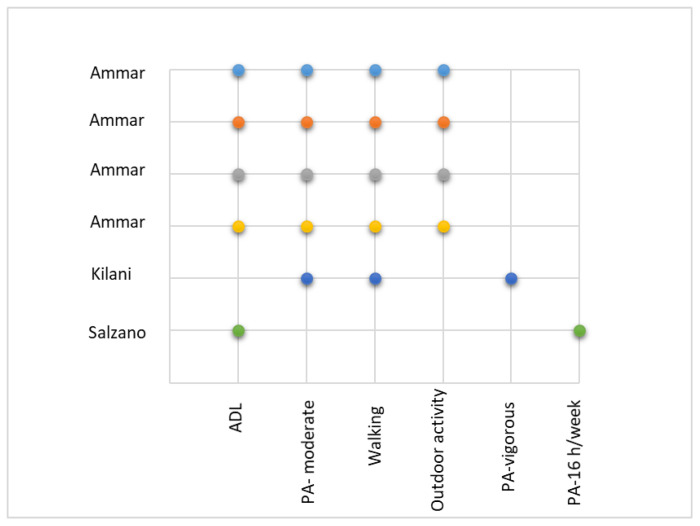
Type of physical activity (ADL: Activity of Daily Living) for healthy people [12,13,14,15,16,17].

The physical activity (PA) has been studied in terms of types of physical activity and is presented in Figure 2.

We illustrate in Table 1, the instruments discussed, the characteristics, the physical activities performed, the interventions and the conclusions derived from the clinical trials studied at healthy adults:

According to Ammar [16], significant increases in uses of the technology allowing the development of PA during home confinement were observed. Mood and feeling has a negative correlation with PA participation, but life satisfaction has a positive correlation with PA. Moreover, the anxiety, depression and other psychological issues during COVID-19 are related with physical activity and its role on the immune function.

Furthermore, Kilani [17] speaks about PA, which could be considered to be a very good predictor of mental wellbeing score and health status, but this depends on PA intensity.

### 3.2. Tools/Variables Which Quantify Motor and Emotional Status at Patients with Neurodegenerative Diseases during Lockdown

Neurodegenerative diseases are pathologies characterized by progressive dysfunction and neuronal damages due to the accumulation of proteins with altered biochemical properties. This process causes changes in neural interconnections affecting movement, speech, memory, intelligence and other brain functions, in accordance with the areas where changes occur in the central nervous system. Neurodegenerative diseases are associated with progressive cognitive and motor decline, having negative social, economic and financial impact, especially as the disease progresses.

In addition to the pathophysiology of neurodegenerative diseases, there are other risk factors that can aggravate and accelerate their evolution, such as lack of physical activity, sedentary lifestyle, weight gain/obesity or the association of other comorbidities (diabetes, atherosclerosis, hypertension). All these impediments to lifestyle, physical activity, eating habits, interpersonal communication and access to public health services were present during the periods of home regimentation imposed by the fight against the spread of coronavirus disease (COVID-19) [19].

As in the case of healthy adults, for patients with neurodegenerative diseases, specific online platforms were developed with questionnaires, semi-structured interviews or other telerehabilitation interventions at home. It was thus possible to quantify the progression of motor impairments induced by the decrease of controlled exercise training led by therapists, and alteration of cognitive status due to the lack of communication and social distance imposed during the period of isolation at home.

The following tools were used to assess the impact on emotional status, cognitive functions impairments, sleeping disorders and quality of life in home confinement: the Mini-mental Scale Examination (MMSE), which evaluates cognitive functions impairments [20,21,22]; the Montreal Cognitive Assessment (MOCA) [23]; the Geriatric -Depression Scale (GDS) [24,25]; Beck Depression Inventory-II (BDI-II), with score from 0 to 63, and high values means an increasing level of depression [26,27]; the Cognitive Emotion Regulation Questionnaire (CERQ) [28]; Social Support (SOZU-K) [29]; the Brief Resilience Scale (BRS) [30]; State Trait Anxiety Inventory (STAI); Optimist-5 point Scale; Quality of Life (QOL); Hoehn and Yahr (HY), Quality of Life short version (SF-8); Mental Component Summary (MCS); Motor experiences daily living (UPDRS) [10]; New-onset/worsening of sleep (NOWS), Restless legs syndrome (RLS), REM Sleep Behavior Disorder (REMBD), Item Content Validity Index (I-CVI), Scale Content Validity Index (S-CVI), Scale content validity Index Universal agreement (S-CVI-UA); Likert Scale; Sleep Disordered Breathing (SDB) [31].

Other variables reflect declining physical activity during lockdown, such as Physical Activity Readiness Questionnaire (PAR-Q), Physical Activity Level (PALs) [19,32], Physical Activity Scale for the elderly (PASE), Physical Activity Level (PALs), Physical Activity Readiness Questionnaire (PAR-Q), Physical Activity Scale for the elderly (PASE) [13,32] and Metabolic equivalents minutes/week (MET) [33].

Other tools can assess signs and symptoms of neurodegenerative diseases related to motor and cognitive dysfunctions well as their changes during the period of social isolation imposed by the spread of SARS infection—Cov-2: for Alzheimer’ Disease; these include the Consortium to Establish a Registry for Alzheimer’s Disease (CERAD-Plus) [19], the Clinical Dementia Rating Scale (CDR) [34], Hamilton Depression Rating (HAMD), Epworth Sleepiness Scale (ESS), Activities Daily Living (ADL) and Neuropsychiatric Inventory (NPI) [35].

Another autoimmune neurodegenerative disease which affects acetylcholine receptors from the level of the postsynaptic membrane of the end plate, Myasthenia gravis, was monitored through MG Quality of Life (MGQOL15), MG Activity of Daily Living (MGADL, Myasthenia Gravis Foundation of America staging (MGFA Scale), Hospital Anxiety and Depression Scale HADS) with a score range between 0–21, where increased values mean worsening depression and anxiety [36].

In Parkinson’s Disease (PD), the neuropathological mechanism entails the loss of pigmented dopaminergic from the brain nuclei belonging to the black substance of the midbrain with production of atypical proteins, called Lewy bodies, subsequently altering the cortico-thalamo-cortical pathways with pathological consequences on the motor and cognitive behavior of the body. Therefore, motor deficiencies such as tremor, bradykinesia, gait disorders and stiffness as well as cognitive impairments, could be quantified through the Personalized Parkinson Project (PPP) [37]; Perceived Stress Scale (PSS) [38]; Unified Parkinson’s Disease Rating Scale (UPDRS); Parkinson Anxiety Scale (PAS); Ruminative Response Scale (RRS) [4]; Physical Component Summary (PCS); Patient global impression of change Scale (PGIC), a scale with a score between 1 to 7, which investigates changes in motor and non-motor disorders in Parkinson’s Disease; Hospital Anxiety and Depression Scale HADS) [10] and Sleep-Scales for Outcomes Parkinson’s Disease (SCOPA-sleep) [39].

In four papers, monitoring patients with MCI has demonstrated the importance of intervention in various ways of physical activity delivered through online platforms, telephone interviews, or with dedicated applications on mental, affective and motor status. So, for 12 older adults of which six women and six men, with mild cognitive impairment, each carrying a polar heart sensor network, and for 8 weeks participated in a fitness and dance training program (two sessions of 90 min/week) delivered through the DIADEM platform, demonstrated improved cardiac performance, benefits in mental functions and enhanced motor status with better walking speed and step length. Monitoring was performed by heart rate recordings and by using the following instruments: MMSE, PAR-Q, GDS and CERAD-Plus [19]. During the pandemic lockdown, such interventions can efficiently compensate for the detraining due to combating the spread of coronavirus disease, while maintaining adequate muscle tone and motor skills.

For 177 older adults with mild cognitive impairment (50), Alzheimer’ disease (105) and dementia with Lewy bodies, which performed activities daily living (ADL) stimulated by caregivers, demonstrated delayed mental and physical deterioration. Monitoring was demonstrated by evaluation of the scores of MOCA, NPI, HAMD, and ESS tools [35]. Another study investigated by telephone semi-structured interview, 4710 patients of which 2809 female and 1901 male, split in 2355 pairs with caregivers, with Alzheimer’ disease, Dementia with Lewy body disease, Frontotemporal dementia and Vascular dementia, which performed activities daily living (ADL). Monitoring was performed through CDR. In this situation, increased caregivers stress risk has been estimated for patients, due to clinical features, lifestyle, dislike of continuity in medical care and deterioration of mental and emotional status of patients with dementia during restrictions imposed by spread SARS-CoV-2 infection. A prospective study discusses the importance of continuing physical activity at home during isolation in the COVID-19 pandemic. This includes moderate effort activity for 150 min/week, 75 min. of sustained effort/week and a strength training intervention provided by an app-based workout with online partners. The measurement of beneficial effects could be carried out through MET and the study includes participants of over 40% Latin America female, Caribbean female and other males and females in developed countries [33].

In another paper, 38 patients with moderate Myasthenia Gravis (not associated with COVID-19), were treated with prednisolone and azathioprine, and physical exercise training consisting of walking, yoga and moderate physical activity delivered through online platforms. Evaluation was carried out by specific tools: MGFA, MGQOL 15, MGADL, HADS and PSQI, which demonstrated worsening wellbeing life quality, anxiety and depression during home confinement [36].

In seven other studies, the evolution of patients with Parkinson’s Disease in the restrictive period imposed by the COVID-19 pandemic was followed. A total of 88 older adults with PD were divided into an experimental (45) and control group (43). Phone interviews were carried out about ADL during lockdown using the specific tools: PASE, HADS and PALs. Evaluation revealed worsening motor impairments with increasing dyskinesia, tremor, freezing of gait, instability stance, muscles pain, rigidity and cognitive disorders, which were augmented sleep dysfunction, depression, anxiety, feeling stressed, lack of concentration and attention [36]. In another paper, investigations were carried out in 832 patients with PD, who were performing moderate walking training for one hour/day. The authors discuss the increasing motor and non-motor impairments during isolation at home assessed by RLS, REMBD, NOWS, I-CVI, S-CVI, S-CVI-UA, Likert Scale, VAS and the SDB scale. Through following these instruments, changes were evaluated in sleep quality, degrees of depression, anxiety, mood and poor life quality [31].

Another study with 100 older adults (45 women and 55 men with PD in the experimental group) and 100 caregivers, were assessed through online questionnaires about physical activity during the imposed home isolation required to combat the spread of COVID-19. The variables used showed, in particular, the alteration of the MCS score in females with PD due to weight loss, lack of exercise (37%) or unhealthy behavioral habits; for caregivers, there were serious concerns (47%) regarding the decrease in PCS score, through increasing stressors, smoking or changes in emotional and affective status during lockdown [10].

In a preliminary study on the stay at home mandate, a self-questionnaire was carried out with 36 patients by mobile based-neurocognitive assessment with PD, of which 53.6% were female and 46.4% were male, regarding physical activity/week, number of active days and average time allocated for physical exercise. Motor and neurocognitive patterns were quantified and performed by using the scales: MTA, MCA, NFI, SWCT and MMSE, which found decreases in motor skills, speech neurocognitive tasks and wellbeing life quality in approximately 80% of the patients with PD [40].

Another paper investigated the impact of the stay at home mandate for 517 adults with PD subject to an international online study, carried out over the course of 12 weeks, in 14 languages with a 64-item questionnaire. The Parkinson’s patients were interrogated on online platforms about weekly physical activity from light physical activity to vigorous physical training and were assessed through SWEMWBS (7–35), PSQI and IPAQ-SF scales. The results showed declining motor functions, quality of sleep, negative feelings and worsening wellbeing of emotional and affective conditions. However, unlike young counterparts, there was greater resistance to stressors during lockdown and greater emotional resistance to older adults with PD [41].

The association of an external stressor, such as the COVID-19 pandemic, has worsened the motor and cognitive symptoms shown with 358 Parkinson’s patients (38.5% female and 62.5% male) who participated in an online study with cognitive and psychological measurements. Patients participated in physical activity for 4 h/week, and their evaluation through PPP, PSS, PAS, RRS, BRS, CERQ, MoCA, SCOPA-sleep, STAI and BDI-II demonstrated worsening motricity with augmentation tremors, rigidity, pain, instability balance, and impairments of gait, but also aggravation of mental, emotional and sleep disorders [39].

The impact of the COVID-19 lockdown was evaluated with a web-based survey for 142 PD patients, of whom 41% were female and 50% were male, belonging to a community dwelling. They performed ADL and walking training. The instruments used for evaluation in quarantine were PAM-13 (0–100 score), which is a validated self-reported questionnaire measuring confidence, self-management, motor skills and cognition. Evaluation using a four-point Likert Scale demonstrated negative impact for 37.3% of the cases. The need for social care for these patients was found to be effective, especially due to the period of restrictions imposed by the COVID-19 pandemic, as approximately 24.8% of participants need caregivers [42].

The literature reveals substantial information about types of PA that were agreed upon by patients with neurodegenerative diseases.

#### Physical Activity in Neurodegenerative Diseases (MCI, AD, DLB, MG FTD and PD)

**Figure 3:**
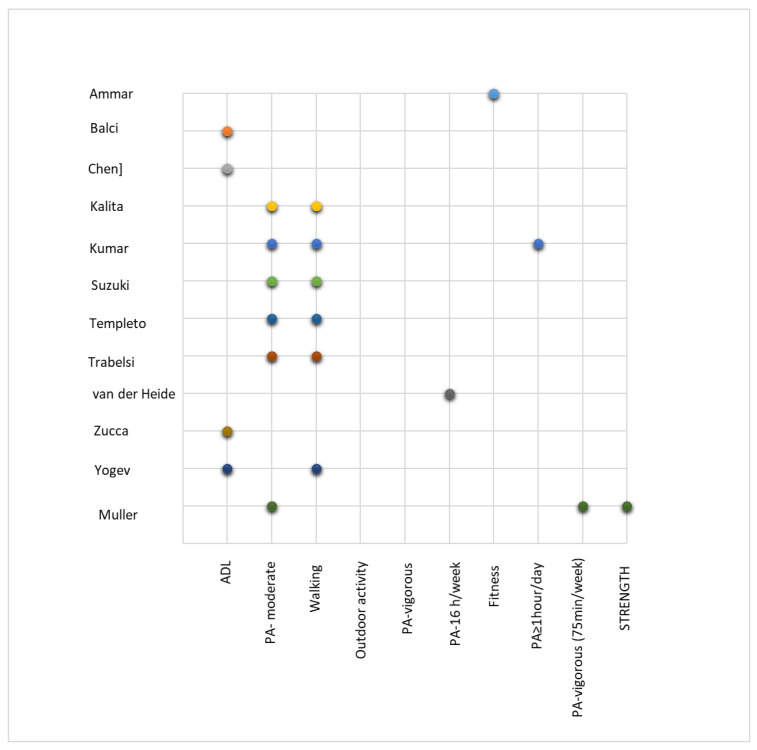
Type of physical activity for patients with neurodegenerative diseases [9,13,26,30,31,32,33,34,35,38,39,41].

Physical activity (PA) has been studied in terms of types of physical activity and is presented in Figure 3.

Table 2 presents the tools discussed, the characteristics, physical activities performed, interventions and conclusions derived from the studies on patients with neurodegenerative diseases.

The relationship between PA and anxiety and depression levels has also been a problem in the pandemic period for PD patients. In this period, PA has experienced substantial changes and even leisure times, household and activities over the course of one week could improve PA levels.

Another study found the lockdown and consequent reduced PA was shown to increase anxiety in more than 68.9% of PD patients, aged 65, who showed preference for household activities [32].

Chen [35] took into consideration the relationship between PA and cognitive, neuropsychiatric symptoms and observed that PA had the most important decrease during one year with a DLB group of patients (*p* ≤ 0.001). At the same time, the PA level showed a sudden decrease and was correlated with the MMSE score.

The impact of COVID-19 for PD patients could be approached in relation to screen time and PA (less than 1 h/day, or more than 1 h/day). PA of more than 1 h/day has been shown to protect against sleep disorders [35].

Motor and non-motor symptoms are related to PA in PD patients; PA involves a decrease in symptoms by 50% for PD patients [40]. PA influences mental wellbeing [41] by improving mood and physical health. The COVID-19 pandemic has created the situation in which low PA can exacerbate cognitive issues in neurodegenerative diseases. This research found moderate PA and walking to decrease by 22–26% during lockdown and the sedentary activity led to sitting increasing by 27.2%.

Thus, the PA and sleep could be considered a predictor for wellbeing and would be interesting to study this in the long term effect.

PA in PD patients has been shown to decrease during COVID-19 and aggravate motor symptom and psychological distress. This could be correlated with worsening of PD symptoms severity and perceived stress [39].

For these patients, the authors confirm that 46.6% of respondents were less active during the pandemic period, but surprisingly, there was no correlation found between time of PA and perceived stress (Pearson correlation R = 0.07, *p* = 0.195).

Yogev [42] discuss the concept of activation in case of PD. This concept also includes PA, and they observed that people who had the highest levels in activity also had an excellent approach to self-management.

The study revealed that more than 67.8% of PD patients reported worsening of symptoms due to cessation of PA, but in the cases of high levels in activity, 69.7% of patients exercised 3–5 times/week or every day using an online application. The PA included stretching, yoga and stationary biking.

The authors concluded that two-thirds of respondents declared a worsening of symptoms, of which, PA was the principal factor.

Müller [33] carried out an analysis of the relationship between COVID-19 and physical activity and observed PA to decrease by 20% and sitting time increased by over 28%. He also took into consideration the anti-inflammatory effect of PA and the reduced effect of IL6 (interleukin 6) that may increase the fight against viral diseases, such as COVID-19.

In this context, exercise prescription should be revised to develop particular aspects such as endurance, resistance, balance exercises or outdoor activities.

This aspect is more important due to the consequences including risk of several chronic diseases, much more because COVID-19 has several metabolic and cardiopulmonary sequels, and at the same time, aggravates depression and dementia.

## 4. Discussion

The purpose of our qualitative screening was to demonstrate the negative impact on the motor outcomes and on the emotional and psychological functions [3] of the isolation period at home as well as the subsequent restrictions imposed by the fight against the spread of the new coronavirus infection on healthy adults but also of patients with neurodegenerative diseases such as: Parkinson’s disease, Alzheimer’ disease, Lewy body disease, and associated diseases (Vascular dementia, Myasthenia gravis), Frontotemporal dementia [6].

Through technological communication systems, such as intervention smartphones and online platforms (Google online, Instagram, Facebook, Tik Tok, Snapchat, WhatsApp, ResearchGate, Twitter, LinkedIn) semi-structured questionnaires had been delivered, online interviews in surveys in multiple languages [13,14,15,16].

In the largest online study for the COVID-19 outbreak, ECBL was conducted in Africa (40%), Asia (36%) and Europe (21%), before and after lockdown, and discussed in four trials [13,14,15,16], in multiple languages. The physical activities performed included exercise classes, or gym classes, walking training, workouts of different intensity (slow, moderate or vigorous intensity, outdoor and indoor physical activity and ADL). The results obtained were interpreted by IPAQ-SF and STBQL and demonstrated in general, declining ongoing weekly physical activity in terms of duration, power and endurance. In terms of ADLs, these were moderate in frequency and velocity [13,14,15,16].

However, there have been challenges in supporting physical activities at home, organizing online exercise groups for performing exercise patterns delivered through questionnaires or telerehabilitation interventions, including training with different intensities and that which is ongoing (such as carried out weekly) as well as in providing support [8,19].

Another study showed that increase of daily physical activities in young people with the diversification of actions, especially food-related actions, allowed for improvement in the diets for adolescents in lockdown compared to the period of direct participation in social life [18].

The most significant changes were related to accentuation, the cognitive function disorders and emotional status, impaired wellbeing quality of life, sleep and unhealthy eating behaviors. The quantification of the outcomes was measured by the instruments: SWEMWBS, SMFQ, SLSQOL, SSPQOL, PSQI and SDBQL, which reported worsening wellbeing and satisfaction of life, increasing mental tensions related to quality of life, depression symptoms, anxiety, lack of communication, enhanced sleep impairments, mental disorders and bad feelings [2,5,9,13]. More pronounced changes in emotional status and mental wellbeing were found especially in women.

Most of the mental and emotional disorders occurred as a result of the cessation of professional activities, lack of communication and socialization, imposed by combating the spread of the SARS-CoV-2 infection by respecting the rules of social distancing.

Along with these came unhealthy eating habits, with the increase in the number of daily meals, rich in fat, carbohydrates and low in protein, excessive alcohol consumption and smoking, which led to weight gain, increased functional disorders and exposure to future morbidity [5,6,25].

Patients with neurodegenerative diseases carrying out different types of physical activities: dance and fitness training, yoga, walking workout, light, moderate or high intensity aerobic exercise and daily activities for living, showed improvements in cardiovascular function, physical performance of the gait pattern, speed and length of the step and balance as well as delays in the decline of motor skills [10,23,35].

Regarding PD patients with improved cardiovascular function, the physical performance of the gait pattern, the dysfunctions of the four cardinal points of the disease, namely, tremor, rigidity, bradykinesia and postural instability, have been shown to be delayed in their evolution by sustained and correctly performed physical exercises [10,20] with instructions from online platforms, and in some situations, supervised by specialist therapists through video applications.

However, in patients with Alzheimer’s disease [33], Lewy body dementia [34], or Frontotemporal dementia and associated Myasthenia Gravis or those with Vascular dementia, who no longer received institutionalized care with therapists but caregivers at home or through information and communication technology that delivered instructions with physical activity programs, there were obvious declines in motor dysfunctions. In these situations, caregivers at home have also been observed to worsen their motor performance by decreasing the physical training and the motor rehabilitation patterns that were imposed on their patients and in which they directly participated.

On the other hand, apart from the degradation of the motor functions, the most significant dysfunctions were registered in the sphere of the cognitive functions and of the neuropsychic, affective and emotional status of both patients and their caregivers [38].

Moreover, the marked disturbances in the sleep/wake circadian rhythm as well as the behavioral changes in the home confinement period, determined the progressive decline in quality of lifestyle, satisfaction and mental wellbeing as affective disorders with depression, sadness, anxiety and feelings of loneliness that reflected lack of socialization from organized communities to emotional rehabilitation programs [40,41]. In addition to COVID-19 as a secondary stressor to the primary stressor, cognitive dysfunction, negative emotions, frustration and mental disorders had been exacerbated.

The cognitive tools through their scores, used clearly, showed an increase in neuropsychiatric symptoms with worsening of depressive symptoms, anxiety and reduced mental wellbeing and speech neurocognitive tasks (MMSE, MoCA, GDS, PASE, HADS, QOL, etc.) [14,15].

**Figure 4:**
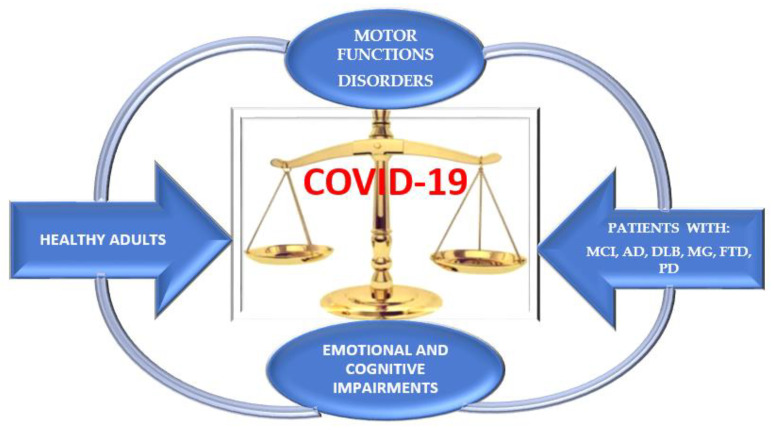
Dynamic of motor and cognitive impairments in healthy adults and patients with neurodegenerative diseases.

Impairment of motor and cognitive status has been shown to be similar, in both healthy adults and patients with neurodegenerative pathology during isolation at home (Figure 4).

The external stressor represented by the COVID-19 pandemic is secondary for patients with neurodegenerative diseases because in terms of neuropathological mechanisms, they are more resistant to the negative impact on cognitive functions and for healthy adults, emotional, affective and neurocognitive functions are more important [27]. Decreasing physical activities affects both types of participants equally, causing impaired motor function in healthy people and the worsening of neuromotor symptoms in patients.

By carefully processing the studies approached, we have found that through the intervention of physical activity, beneficial results are obtained on both motor and neurocognitive functions.

The use of online technology and tools that can measure vital functions of motion sensors that quantify physical activity at home has had a considerable advantage in home confinement due to remotely monitoring the varying degrees of dysfunction in healthy adults and the progression of neurodegenerative diseases [32,39].

However, the use of online e-learning platforms that provide standardized information, programs and questionnaires that help monitor the motor and cognitive coordinates of healthy adults and patients with neurodegenerative diseases, is conditioned by limited access to these technologies, costly financial commitments as well as the educational level of the participants who can understand the foreign language and apply these models.

## 5. Conclusions

By applying semi-structured questionnaires, intervention smartphones or online interviews, telerehabilitation has been found to be easier and can address very large communities.

Ongoing weekly activities, in terms of duration, power and endurance, have been found to be declining in the pandemic. In terms of ADLs, these were moderate in frequency and velocity.

Accentuation in cognitive function disorders and emotional status, impaired wellbeing quality of life and sleep and unhealthy eating behaviors have all been observed.

The widespread use of tools capable of assessing and monitoring the degree of motor and cognitive dysfunction has led to the development of new rehabilitation and recovery strategies in terms of changes in the physical and emotional status of healthy adults and patients with neurodegenerative diseases.

For patients with neurodegenerative diseases, the physical activities of aerobic type improved cardiovascular function and the physical performance of the gait pattern.

Specific symptoms, such as remor, rigidity, bradykinesia and postural instability, have been delayed in evolution in the conditions of sustained and correctly performed physical exercises with instructions from online platforms.

The cognitive tools, through their scores, clearly showed an increase in neuropsychiatric symptoms with worsening of depressive symptoms, anxiety and reduced mental wellbeing and speech neurocognitive tasks.

The use of online technology has had a considerable advantage during home confinement due to its ability to remotely monitor the varying degrees of dysfunction in healthy adults and the progression of neurodegenerative diseases.

New health policies that support good mental, physical and quality of life should be supplemented as future perspectives.

In this sense, it is necessary to participate as widely as possible in educational programs developed by specialists, applied to the healthy population, people with special needs and their caregivers, to promote a healthy lifestyle by increasing participation in organized physical activities, increasing socialization, maintaining mental balance and healthy eating behaviors, delivered either directly or through the participation of online technology.

It is necessary for state governments to be involved and to create new policies for training caregivers with financial support for new recovery and rehabilitation strategies. Thus, it will be possible to increase the access for other categories of staff who can be easily trained and understand the techniques of care at home or in assisted communities.

The importance of an interdisciplinary strategy that includes the promotion of physical activity and encouraging use of technology for PA participation should be stressed.

### Limitations and Advantages of the Study

The limitations of this study include that the possible subjective nature of the information presented and the relatively small number of the studies that satisfied our selection criteria.

The advantages of this study include its approach to complex issues on the impact of the COVID-19 pandemic lockdown for both healthy people and neurodegenerative patients. The study provides new information based on the literature research, which could be used for the creation of new health policies and future perspectives.

## Figures and Tables

**Table 1:** Summary of the characteristics, physical exercise, main tools and interventions with healthy adults.

Authors	Individuals	Characteristics	Age/Gender	Physical Exercise	Tools	Interventions	Conclusions
Ammar A. et al. (2020) [14]	1047 individuals	- ECBL-COVID-19—online survey multi-languages - AHCL—Active and Healthy confinement lifestyle - before and during COVID-19	Adults 54% female 46%—male 36%—Asia, 40%—Africa, 21%—Europe, 3%—other	- ADL - Exercise class - Gym class	- SWEMWBS (1–35) - SMFQ (0–26) - SLSQL - SSPQOL - IPAQ-SF - SDBQL - PSQI - STBQL	- Online platforms: - Mail - WhatsApp - Facebook - ResearchGate - Twitter - LinkedIn	- Decreasing mental wellbeing in home confinement, - Increasing depression symptoms (≥12) - Changing in sleep quality, satisfaction life, physical activity, social participation, - Enhancing mood and bad feelings.
Ammar A. et al. (2020) [15]	1047 individuals	- ECBL-COVID-19 -online survey multi-languages - before and during COVID-19	Adults 54% female 46%—male 36%—Asia, 40%—Africa, 21%—Europe, 3%—other	- Physical exercise with vigorous, moderate intensity training - Walking activity	- SWEMWBS - SMFQ - SLSQL - IPAQ-SF - SDBQL (0–15) - PSQI - -STBQ-L	- Google online	- Declining of duration time for weekly physical activity, - Increasing negative impact for diet behaviors in the sense of augmentingn snack consumptions between meals, enhancing alcohol intake, increasing the number of daily meals;an unhealthy diet is important indicator for sedentary lifestyle.
Ammar A. et al. (2020) [13]	1047 individuals	- ECBL-COVID-19 -online survey multi-languages - before and during COVID-19	Adults 54%—female 46%—male 36%—Asia, 40%—Africa, 21%—Europe, 3%—other	- ADL	- SWEMWBS - SMFQ - SLSQL - IPAQ-SF - SDBQL - PSQI - STBQ-L - SSPQ-L (14–70) - SLSQL (3–21)	- Online platforms: - Mail - WhatsApp - Facebook - ResearchGate - Twitter - LinkedIn	- Decreasing friends, neighbors direct intercommunication in lockdown period with strongly mental implication for individuals, - Developing socialization on online communication platforms.
Ammar A. et al. (2020) [16]	1047 individuals	- ECBL-COVID-19 -online survey multi-languages - before and during COVID-19 - Cardiovascular risk	Adults 54%—female 46%—male 36%—Asia, 40%—Africa, 21%—Europe, 3%—other	- Outdoor or indoor physical activity	- SWEMWBS - SMFQ - SLSQL - IPAQ-SF - SDBQL - PSQI - STBQ-L - SSPQ-L - SLSQL	- Online platforms: - Mail - WhatsApp - Facebook - ResearchGate - Twitter - LinkedIn - Smart phone - Watch	- Disrupting wellbeing and satisfaction life, communication, - Developing mental disorders, anxiety, sadness, mood, states of accentuated nervousness during quarantine. - Increasing tensions for individuals which were retired or unemployment comparative with before lockdown period when they were workers, - Encouraging wellbeing for promoting a healthy lifestyle by participating in physical activities at home or in organized groups keeping the rules of distance imposed by the pandemic and hygiene.
Kilani H.A. et al. (2020) [17]	1723 participans	- Arabian communities - Health status - Mental wellbeing - Dietary behavior - PA - Sleep quality	32.2 years 806-female 37.4 years 917-male	- Walking - Moderate physical activity - Vigorously activity - MET	- lower mental score - female - good dietary quality - sleeping score - FFQ - IPAQ - WHO-5 - PSQI	- Online Google - WhatsApp - Facebook - ResearchGate - Twitter - LinkedIn	- Decreasing mental wellbeing at female against male, - Increasing sleep quality at both gender corelated with good quality diet, - Significant improving mental health, cognition functions in the conditions of maintaining a sustained physical activity such as: walking, moderate and vigorous physical exercise.
Salzano G. et al. (2021) [18]	1860 youth	- Lower secondary school - Upper secondary school	12–18 years Female—61.7% Male—38.3%	- Physical activity (1 h-6 h/week) - ADL (cooking, gardening, household activities)	- Average number of text or chat messages/100 day	- Web-based survey - Personal computers - Smartphones - Tablets - Instagram - Facebook - Tik Tok - Snapchat	- Increasing physical activity indoor during lockdown - Good mental resistance in stay-at-home mandate with skills acquiring of new skills - Altering circadian rhyme sleep/wake due to intense use of media platforms for intense global socialization and the maintenance of friendships and emotional status on social networks - Increasing anxiety, depression and other psychological disorders through excessive use of social networks that can promote cyberbullying and sexting - In terms of eating, no unhealthy eating habits were acquired due to the lack of training in the communities and the consumption of small meals, snacks, sweets, excess juices; on the contrary the promotion of cooked meals and healthier foods at home was observed.

ECBL: Effects of Home Confinement on multiple lifestyle behaviors; SWEMWBS: Short Warwick–Edinburgh Mental Wellbeing Scale; SMFQ: Short Mood and Feelings Questionnaire; SLSQL: Short Life Satisfaction Questionnaire for Lockdowns; SSPQOL: Short Social Participation Questionnaire for Lockdowns; IPAQ-SF: International Physical Activity Questionnaire Short Form; SDBQL: Short Diet Behaviors Questionnaire for Lockdowns; PSQI: Pittsburgh Sleep Quality Index; STBQL: Short Technology-use Behaviors Questionnaire for Lockdowns; SSPQ-L: Short Social Participation Questionnaire-Lockdowns; SLSQ-L: Short Life Satisfaction Questionnaire for Lockdowns; EQ-5D-5L: Euro QoL-5 dimensions; ADL: Activity of Daily Living; HADS: Hospital Anxiety and Depression Scale; FFQ: Food Frequency Questionnaire; WHO-5: World Health Organization—wellbeing score.

**Table 2:** Summary of the characteristics, physical exercise, main tools and interventions for patients with neurodegenerative diseases.

Authors	Individuals	Characteristics	Age/Gender	Physical Exercise	Tools	Interventions	Conclusions
Ammar A., et al. (2021) [14]	12 individuals	- MCI (mild cognitive impairment)	Older adults 6—female 6—male	- 8 weeks fitness training dance training - 2 sessions/90 min/week - DIADEM training program	- HR-Heart Rate - MMSE (24–27) - PAR-Q - GDS - CERAD Plus	- Polar heart rate sensor (H10) - Wireless polar team	- Improving performance cardiac at patients with MCI after physical exercise training, - Increasing physical performance regarding speed gait and length of step, - Continuing home-based physical training in social distancing rules during lockdown with important benefits for mental and physical functions.
Balci B., et al. (2021) [32]	88 individuals - experimental group (*n* = 45) - control group (*n* = 43)	Parkinson’s Disease (PD)	Older adults	ADL	Increasing: - Tremor - Dyskinesia - Rigidity - Freezing of gait - Instability postural - Sleep impairments - Muscle pain - PASE - HADS - PALs	- Phone interview	- Augmentation of non-motor and motor functions at PD patients during quarantine, - Increasing anxiety and depression through distancing, detraining and worse socialisation during confinement home.
Chen Z.C., et al. (2021) [33]	105-AD 50-MCI 22-DBL	- MCI - Alzheimer’ disease (AD) - DLB-dementia with Lewy body disease	Older adults	- ADL	- MMSE - MOCA - NPI - HAMD - ESS	- CT scans - MRI - PET-positron emission tomography	- During lockdown and stay at home, caregivers should help patients with cognitive impairment and dementia to maintain exercise routines training home based of a certain intensity and frequency and to maintaining socialisation with friends and relatives by phone and another network. - Telerehabilitation should be one of the most advantageous interventions during quarantine to prevent the evolution of mental and physical impairment in patients with MCI.
Kalita J., et al. (2021) [36]	38 individuals with MG (myasthenia gravis) non COVID-19	- MG - Treatment with prednisolone and azathioprine	45 yrs	Physical exercise training: - yoga - walking - moderate physical activity	- MGFA - MGQOL15 (0–4) - MGADL - HADS (0–21) - PSQI	- Phone interviews - Whats app.	- Decreasing quality of life through detraining of physical activity, - Aggravation of physical and mental dysfunctions of patients with MG in the conditions imposed by lockdown.
Kumar N., et al. (2021) [31]	832 individuals	- PD-Parkinson disease ˃7 years - Home confinement - questionnaire - Sleep disturbance - Features suggestive of RLS - Features of REMBD - Sleep disorders - Worsened motor features - Worsened non-motor features	>50 years	- walking - moderate physical activity - ˃1 h/day	- RLS - REMBD - NOWS - Likert Scale - I-CVI - S-CVI - S-CVI-UA - VAS - SDB	- Phone - Screen time	- During COVID-19 pandemic, quarantine determined the worsening of sleep disorders at all PD’ patients, - Increasing motor and non-motor impairments due to lacking physical activity, poor life quality, sleep disorders.
Suzuki K., et al. (2021) [10]	- experimental group- - (*n* = 100 PD) - control group-caregivers (*n* = 100 caregivers)	- Parkinson’s disease - HY (2/3) - Comorbidities - Motor and cognitive impairments	>72 years 45-male 55-female	- physical activity	- PGIC (1–7) - QOL - HADS - PCS - MCS - SF-8	Online platform with delivering questionnaires	- Increasing severity signs and symptoms of PD’ patients regarding pattern gait, rigidity, tremor and disturbances sleep, - Enhancing anxiety, depression, mood, attention impairments at PD, - Decreasing physical activity in lockdown had negative impact for daily and walking activities, being associated with important motor disorders.
Templeton J.M., et al. (2021) [40]	28 patients—self-questionnaire 8 patients—mobile based neurocognitive	- Parkinson’s disease - Self-reporting - Functional assessment	>52 years - 46.4%—male - 53.6%—female	- Physical activity/week - number of active days on week - average time physical activity	- Number of active days - Number of active minutes - Number of activities - Average temporal metrics - Likert Scale (1–5) - MMSE - MCA - MTA - SWCT - NFI	- Self-reporting questionnaire - Mobile -based neurocognitive measurements	- Decreasing controlled physical activity in stay at home mandate (SaHM) caused worsening, moderately or higher, of at least one PD symptoms individuals (80%), - Increasing interval by at least two times in which the objective actions are completed, - Reducing wellbeing lifestyle, motor and speech neurocognitive tasks in home confinement.
Trabelsi K., et al. (2021) [27]	517 individuals	- Parkinson disease - International online survey - (64 items)—12 week in 14 languages - PA - Diet - Sleep - Social participation - Psychosocial - support	>55 years	- -Weekly physical activity - (walking training low, moderate and vigorous physical exercise, MET)	- SWEMWBS (7–35) - PSQI - IPAQ-SF	- Online platforms	- Keeping constant on mental wellbeing during stay at home mandate, whereas elderly patients with PD are more emotionally resistant being more accustomed to stressors than young counterparts, - Decreasing level homework activities because of lack of physical exercise, which means worsening motor functions, - Declining sleep quality in lockdown and the appearance of other disturbances which induced negative emotions and more frustration.
van der Heide A., et al. (2020) [39]	358 individuals	- PD - Collection blood, stool and cerebrospinal fluid - MRI	>53 years - Male (62.5%) - Female (38.5%)	˃4 h PA/week	- UPDRS - anxiety - stressor load - personality features - PPP - PSS - PAS - RRS - BRS - CERQ - MoCA - SCOPA-sleep - STAI - BDI-II	- Google platforms - Online survey (motor, cognitive and psychological measurements)	- Worsening motor symptoms such as tremor, rigidity, pattern gait, pain and postural instability, - Increasing change in emotional status induced by stressor load related COVID-19 outbreak, - Enhancing neuropsychiatric symptoms such as: depression, anxiety, mood, ruminations through decreasing physical activity.
Zucca M., et al. (2021) [34]	4710 (2355-pairs) - Caregivers - Patients with dementia	- Mild dementia - (AD, DLB, VaD, FTD) - Stress symptoms - (anxiety, irritability, overwhelmed, anguish, - abandonment)	>46 years Male—1901 Female—2809	ADL	- CDR	- Phone semi-structured interview	- Increasing stress symptoms (one symptom—90% or more symptoms—30%) of caregivers with dementia patients linked of consequences COVID-19, - Enhancing conflicting relationship and discontinuity in assistance for female caregivers with dementia patients in time lockdown.
Yogev-Seligmann G., et al. (2021) [42]	142 individuals	- PD - Lockdown - Community dwellings	>63 years Male—59% Female—41%	- ADL - walking training	- Current functional status - Health - Medical care - Wellbeing in quarantine - PAM-13 (0–100) - Four-point Likert Scale	- Web-based survey	- Decreasing walking ability in 37.3% of cases, - Worsening of motor and cognitive disfunctions at PD’ patients (43%), - increasing the need for care assistance for ADL (24.8%), - Enhancing neuropsychiatric symptoms such as depression, anxiety, tired, moody and loneliness (42%) - Discontinuity of rehabilitation treatments.
Müller P., (2020) [33]	˃40% Latin America women ˃40% Caribbean women ˃40% male and women developed countries.	- Vascular dementia (AD)	>40% women >40% male	- 150 min moderate intensity effort physical/week - 75 min vigorous intensity effort physical/week - strength training/week	- MET - WHO	App-based training with online partners	- Worsening physical activity during isolation at home imposed by COVID-19 with negative results about primary and secondary vascular dementia, - Decreasing outdoor physical exercise represent a major problem of health public for people with Alzheimer’ disease.

SWEMWBS: Short Warwick–Edinburgh Mental Wellbeing Scale; SMFQ: Short Mood and Feelings Questionnaire; SLSQL: Short Life Satisfaction Questionnaire for Lockdowns; IPAQ-SF: International Physical Activity Questionnaire Short Form; SDBQL: Short Diet Behaviors Questionnaire for Lockdowns; PSQI: Pittsburgh Sleep Quality Index; STBQL: Short Technology-use Behaviors Questionnaire for Lockdowns; SSPQ-L: Short Social participation questionnaire-lockdowns; MMSE: Mini-mental Scale Examination; PAR-Q: Physical activity Readiness Questionnaire; GDS: Geriatric Depression Scale; CERAD Plus: Consortium to Establish a Registry for Alzheimer’s Disease; MOCA: Montreal Cognitive Assessment; NPI: Neuropsychiatric Inventory; HAMD: Hamilton depression rating; ESS: Epworth sleepiness scale; EQ-5D-5L: Euro QoL-5 dimensions; ADL: Activity of Daily Living; PASE: Physical Activity Scale for the elderly; HADS: Hospital Anxiety and Depression Scale; PALs: Physical Activity Level; MG: Myasthenia Gravis; MGQOL15: MG Quality of life; MGADL: MG Activity of Daily Living; MGFA Scale: Myasthenia Gravis Foundation of America staging; FFQ: Food Frequency Questionnaire; WHO-5: World Health Organization—wellbeing score; MET: Metabolic equivalents minutes/week; RLS: restless legs syndrome; REMBD: REM sleep behavior disorder; NOWS: New-onset/worsening of sleep; I-CVI: Item content validity Index; S-CVI-Scale Content Validity Index; S-CVI-UA: Scale Content Validity Index Universal agreement; VAS: Visual Analog Scale; SDB: Sleep Disordered Breathing; PGIC: Patient Global Impression of Change Scale; QOL: Quality of life; PCS: Physical Component Summary; MCS: Mental component summary; SF-8: Short form; HY: Hoehn and Yahr stage; Likert Scale; MCA: Montreal Cognitive Assessment; MTA: Menu Task Assessment; SWCT: Stroop Word Color Test; NFI: Neurobehavioral Functioning Inventory; PPP: Personalized Parkinson Project; PSS: Perceived Stress Scale; UPDRS: Unified Parkinson’s Disease Rating Scale; PAS: Parkinson Anxiety Scale; RRS: Ruminative Response Scale; BRS: Brief resilience Scale; CERQ: Cognitive Emotion Regulation Questionnaire; STAI: State Trait Anxiety Inventory; BDI-II: Beck’s Depression Inventory; SCOPA -sleep: Scales for Outcomes in PD; MoCA: Montreal Cognitive Assessment; BFI: neuroticism; VaD: Vascular dementia; CDR: Clinical Dementia Rating Scale; FTD: Frontotemporal Dementia; DLB: Lewy body disease; PAM-13: Patient’s Activation Measure.

## Data Availability

Not applicable.
